# Promoting *Shewanella* Bidirectional Extracellular Electron Transfer for Bioelectrocatalysis by Electropolymerized Riboflavin Interface on Carbon Electrode

**DOI:** 10.3389/fmicb.2018.03293

**Published:** 2019-01-15

**Authors:** Long Zou, Xian Wu, Yunhong Huang, Haiyan Ni, Zhong-er Long

**Affiliations:** College of Life Science, Jiangxi Normal University, Nanchang, China

**Keywords:** extracellular electron transfer, bioelectrocatalysis, riboflavin, *Shewanella*, electropolymerization

## Abstract

The extracellular electron transfer (EET) that connects the intracellular metabolism of electroactive microorganisms to external electron donors/acceptors, is the foundation to develop diverse microbial electrochemical technologies. For a particular microbial electrochemical device, the surface chemical property of an employed electrode material plays a crucial role in the EET process owing to the direct and intimate biotic-abiotic interaction. The functional modification of an electrode surface with redox mediators has been proposed as an effectual approach to promote EET, but the underlying mechanism remains unclear. In this work, we investigated the enhancement of electrochemically polymerized riboflavin interface on the bidirectional EET of *Shewanella putrefaciens* CN32 for boosting bioelectrocatalytic ability. An optimal polyriboflavin functionalized carbon cloth electrode achieved about 4.3-fold output power density (∼707 mW/m^2^) in microbial fuel cells and 3.7-fold cathodic current density (∼0.78 A/m^2^) for fumarate reduction in three-electrode cells compared to the control, showing great increases in both outward and inward EET rates. Likewise, the improvement was observed for polyriboflavin-functionalized graphene electrodes. Through comparison between wild-type strain and outer-membrane cytochrome (MtrC/UndA) mutant, the significant improvements were suggested to be attributed to the fast interfacial electron exchange between the polyriboflavin interface with flexible electrochemical activity and good biocompatibility and the outer-membrane cytochromes of the *Shewanella* strain. This work not only provides an effective approach to boost microbial electrocatalysis for energy conversion, but also offers a new demonstration of broadening the applications of riboflavin-functionalized interface since the widespread contribution of riboflavin in various microbial EET pathways together with the facile electropolymerization approach.

## Introduction

Microbial electrochemical systems (MESs) have become an intensive focus of fundamental and applied research owing to their potential to achieve sustainable value-added resources (bioelectricity, biofuels and chemical commodities) from organic or inorganic wastes (Logan and Rabaey, 2012; Zou and He, 2018; Zou et al., 2018), thus providing a promising solution for the increasingly serious risks of energy shortage and environmental degradation. Electrochemically active microorganisms (EAMs) function as biocatalysts to convert chemical energy to electricity, and vice versa. In MESs, they have extracellular electron transfer (EET) ability to donate or accept electrons to/from an electrode. The electron exchange process at the biotic-abiotic interface is a footstone for developing diverse MESs and is also regarded as a crucial rate-limiting step for overall performance. By virtue of advances in design of device configurations, engineering of electrode materials, transformation of biocatalysts and optimization of operating conditions, the MES performances have achieved great improvement in recent years (Logan et al., 2015; Santoro et al., 2017; Saratale et al., 2017; Katuri et al., 2018), but they are still unable to meet the requirements of real-world applications. For instance, the present electrical power densities of microbial fuel cells (MFCs) ranging from several hundreds to thousands of mW/m^2^ (Do et al., 2018) are still lower than conventional chemical fuel cells by one to two orders of magnitudes. Thus, more efforts are perseveringly needed to be directed to enhance microbial EET rates for boosting MES development and future applications.

In an MES device, the applied electrode directly interfaces with EAM cells in general. Therefore, the chemical properties of electrodes play a crucial role in microbial growth and metabolism as well as their EET processes via the intimate abiotic-biotic interaction (Du et al., 2013). Inspired by this, a range of strategies has been developed to tailor the electrode surface chemistry for fast thermodynamic and kinetic processes, including conventional surface modification via physical and chemical treatment (Kumar et al., 2013) and creative functionalization with diverse redox molecules, conducting polymers, metals and metallic compounds, etc. (Tang et al., 2014; Liao et al., 2015; Zhao et al., 2015; Xu and Quan, 2016; Zou et al., 2017a,b). Taking into consideration the hypothetic EET pathways that refer to direct electron transfer via microbial membrane-bound cytochromes and/or nanowires (i.e., DET) and indirect electron transfer mediated by redox-active small molecules acting as electron mediators (i.e., MET) (Shi et al., 2016; Kumar et al., 2017), the functionalization of an electrode with proper electron mediators could be a powerful approach to improve interface electrochemical activity for microbial electrocatalysis. Notably, *in situ* immobilization of electron mediators onto an electrode surface can also circumvent the problems of washing out and possible secondary pollution when these electron mediators are directly added into electrolytes (Wang et al., 2017). Up to now, various artificial electron mediators such as neutral red (Guo et al., 2013), anthraquinones-2-sulfonate (Tang and Ng, 2017) and anthraquinones-2,6-disulfonate (Martinez et al., 2017) have been adopted to modify MES electrodes through either chemically covalent linkage or electrochemical polymerization, while they usually cause some concerns because of possible biological toxicities for these artificial compounds. Alternatively, flavins (endogenous electron mediators excreted from *Shewanella* species) aroused great attention recently on account of their high electron transfer capacity and inherent biocompatibility. In addition, flavins can also function as a bound cofactor of outer-membrane cytochromes to enable fast electron transport (Okamoto et al., 2013), indicating the flexible EET ways for them. As a test, Wang et al. (2017) made use of riboflavin (RF) to modify an electrochemically exfoliated graphene electrode via a simple physical adsorption procedure, and the modified electrode achieved an improved MFC power output. Increased bioelectrocatalytic activity was also observed for an electrode modified with peptide nanotubes encapsulating riboflavin when used as a bioanode and biocathode (Xu and Quan, 2016; Xu et al., 2017), indicating the superiority of riboflavin-functionalized electrodes on bidirectional EET (i.e., outward electron transfer and inward electron transfer). However, these encapsulated riboflavin monomers in the nano-sized tubes were presumably inaccessible for microbial cells by reason of adverse steric-hindrance effect. Although almost all the discovered *Shewanella* strains can self-secrete flavins as their electron mediators for EET processes, the concentration of flavins produced by themselves is, without doubt, not enough to enable robust interfacial electron transfer (Yang et al., 2015). Thereby, the development of an effective and durable flavins-based interface with strong electrochemical activity through a facile and tailorable approach is greatly beneficial to promote MES development.

The technique of electrochemical polymerization could be a simple and achievable approach to immobilize diverse redox-active mediators onto electrode surfaces (Martinez et al., 2017; Naik and Swamy, 2017; Wang and Feng, 2017). In general, this immobilization technology can effectively relieve the loss of electron mediators away from electrodes compared to a simple physical absorption method, thereby endowing great potential for long-term usage. Additionally, a uniformly electro-deposited electron mediator interface can provide a reliable platform to study the underlying effect of redox-active interface on microbial EET pathways and the bioelectroctatalytic process. Herein, polyriboflavin-functionalized carbon electrodes were fabricated via a simple electrochemical polymerization approach and then used to evaluate the roles in boosting the bioelectrocatalytic ability of *Shewanella putrefaciens* CN32 in terms of MFC bioelectricity generation and fumarate reduction. Furthermore, the underlying effect of the electropolymerized riboflavin interface on microbial bidirectional EET process was studied.

## Materials and Methods

### Bacterial and Culture Conditions

Strain *S. putrefaciens* CN32 was purchased from ATCC (Number: BAA-1097), and its mutant strain with in-frame deletion of genes encoding out-membrane cytochromes MtrC and UndA (designated as “*ΔmtrC/undA*” strain) was constructed in our previous work (Wu et al., 2018). All strains were routinely cultivated in Luria-Bertani (LB) broth (10 g/L tryptone, 5 g/L yeast extract (Oxoid, United Kingdom), 5 g/L NaCl, pH 7.2) at 30°C with shaking at 180 rpm to a desired optical density at 600 nm (OD_600_) of 1.5. The bacterial cells were harvested by centrifugation, with 5000 rpm for 10 min and washed with M9 buffer (6 g/L Na_2_HPO_4_, 3 g/L KH_2_PO_4_, 1 g/L NH_4_Cl, 0.5 g/L NaCl, 1 mM MgSO_4_, 0.1 mM CaCl_2_) for three times to remove metabolites, then re-suspended in fresh M9 buffer for inoculation in bioelectrochemical reactors. All chemicals were of analytical grade and were purchased from Aladdin Reagent Co., Ltd. (Shanghai, China) unless otherwise indicated.

### Electrode Preparation, Bioelectro-Reactor Configuration and Operation

Electrochemical polymerizations of riboflavin (RF) onto carbon cloth (CC) electrode were performed as described previously (Radzevic et al., 2016). Typically, a piece of CC electrode (1 × 1.5 cm) purchased from Hong-Kong PHYCHEMi Co., Ltd., China, was firstly polished electrochemically in 0.1 M KCl solution by scanning the applied potential repeatedly between -1.0 and +1.0 V at a scanning speed of 0.1 V/s for at least 15 cycles until constant cyclic voltammograms (CVs) were obtained. Subsequently, the polished CC electrode was successively rinsed with ultrapure water and transferred into 0.1 M phosphate buffered saline (PBS, pH 7.0) solution with the addition of 0.15 M NaCl and 1 mM RF. The electropolymerization of riboflavin was obtained by repeated scanning in the potential window from -1.0 to +1.5 V at a scanning speed of 50 mV/s for 20, 30, and 40 cycles, whereby the cycle counts were termed as PRF@CC-20, PRF@CC-30 and PRF@CC-40, respectively. The prepared polyriboflavin functionalized carbon cloth (PRF@CC) electrode was rinsed with ultrapure water for three times and then dried at 60°C overnight. The CC electrode was immersed into the riboflavin solution for 48 h to achieve spontaneous physical-adsorption and then rinsed with ultrapure water followed by dry for comparison (the prepared electrode was designated as RF/CC). All electrochemical experiments were conducted by using the CHI 760E electrochemical working station (Chenhua Instrument Co., Ltd., Shanghai, China), and all potentials in this work are reported versus saturated calomel electrode (SCE).

The configuration and operation of H-type dual-chamber MFCs were carried out according to our previous reports (Zou et al., 2016a,b). The bacterial cell suspensions in M9 buffer containing 18 mM lactate as electron donors were inoculated into anodic chamber, and the cathodic chamber was filled with 20 mM potassium ferricyanide dissolved in 0.01 M PBS solution as terminal electron acceptors. The output voltage (*V*) across the external load resistance (*R*) of 1500 Ω was recorded by a digital multimeter. The current density (*I*) was calculated using *I* = *V*/*R*, the power density (P) was calculated using *P* = *V* ×*I*. At steady-state of MFCs, the polarization curves were obtained by measuring the stable voltage outputs under different loading of external resistances from 50000 to 400 Ω. Both power density and current density were normalized to the projected surface area of anode area.

The bacterial inward electron transfer from the electrode (cathode) to bacterial cells was evaluated with the electricity consumption rate for the reduction of fumarate to succinate by *S. putrefaciens* CN32 and its mutant strain in three-electrode electrochemical cells (Yong et al., 2014; Si et al., 2015). A platinum sheet and a saturated calomel electrode were used as counter electrode and reference electrode, respectively. The carbon cloth and prepared PRF@CC electrode were used as the working electrode at an applied potential of -0.6 V for comparison. After acclimation under the poised potential for about 30 min, fumarate was added into the reactor with a final concentration of 20 mM for observation of the change of cathodic current input.

### Characterization and Analysis

The surface morphologies of prepared electrode materials and bacterial cells grown on electrodes were observed using scanning electron microscopy (SEM, JEOL JSM-7800F, Japan). The electrodeposited polyriboflavin on the CC electrode was detected by Fourier transform infrared (FTIR) spectrum using a Nicolet 6700 FTIR spectrometer (Thermo Fisher Scientific Inc., United States), and Raman spectrum using a Labram HR800 Raman spectrometer (Jobin Yvon, France). The CV, electrochemical impedance analysis (EIS) and amperometric I-T experiments were performed in a three-electrode electrochemical cell that consisted of a working electrode, a SCE reference electrode and a carbon cloth counter electrode, according to our previous work (Zou et al., 2017b). Analyses were performed without stirring at the ambient temperature. The attached biomass on the electrode was quantified in the form of total protein content by using a BCA Protein Assay Kit [Sangon Biotech (Shanghai) Co., Ltd., China] according to the manufacturer’s protocol. For collection of attached proteins, the bio-electrode from bio-electroreactors was placed in a 1.5 mL tube containing 1 mL of 0.2 N NaOH. The tube was then heated to 96°C for 20 min to lye and detach bacterial cells away from the electrode surface, followed by cooling to ambient temperature for subsequent determination (Coursolle et al., 2010).

## Results and Discussion

### Preparation and Characterization of Polyriboflavin Functionalized Electrode

To prepare the polyriboflavin-functionalized CC electrode, a cleaned CC electrode was placed in 1 mM riboflavin solution for repeated CV scanning. The electropolymerization CV curves (Supplementary Figure S1) showed two pairs of redox peaks at potential of -0.363 / -0.497 V and -0.22 / -0.269 V, respectively. Meanwhile, a sharp oxidation peak at potential of +1.497 V was also observed due to the radical formation of nitrogen or carbon atoms in the isoalloxazine ring of riboflavin. All oxidation peaks grew with increasing numbers of CV cycles, which reflected a previous report (Radzevic et al., 2016) and indicated the gradual growth of polyriboflavin on the CC electrode surface. More detailed mechanisms of electrochemical polymerization of riboflavin can be found in previous reports (Radzevic et al., 2016; Celiesiute et al., 2017), and a possible bonding mode between molecules in the polyriboflavin is shown in Supplementary Figure S2. The FTIR and Raman spectra of the prepared PRF@CC-30, bare CC and pure riboflavin are compared in Supplementary Figure S3. The PRF@CC-30 electrode showed a similar FTIR spectrum as that of the CC electrode, except for a stronger peak in the 3000–3600 cm^-1^ region that is specified as the O–H stretching vibration of hydroxyls. This detectable variation likely originated from the presence of polyriboflavin with abundant hydroxyls attached on the CC fiber surface. The Raman spectrum of the PRF@CC-30 electrode not only revealed the obvious D and G bands similar to the bare CC electrode, but also exhibited some additional small peaks, which could also suggest the presence of electropolymerized riboflavin interface. Furthermore, the high-resolution SEM images (Supplementary Figure S4) verified that the electropolymerized riboflavin takes the form of thin film on the carbon fiber.

**FIGURE 1:**
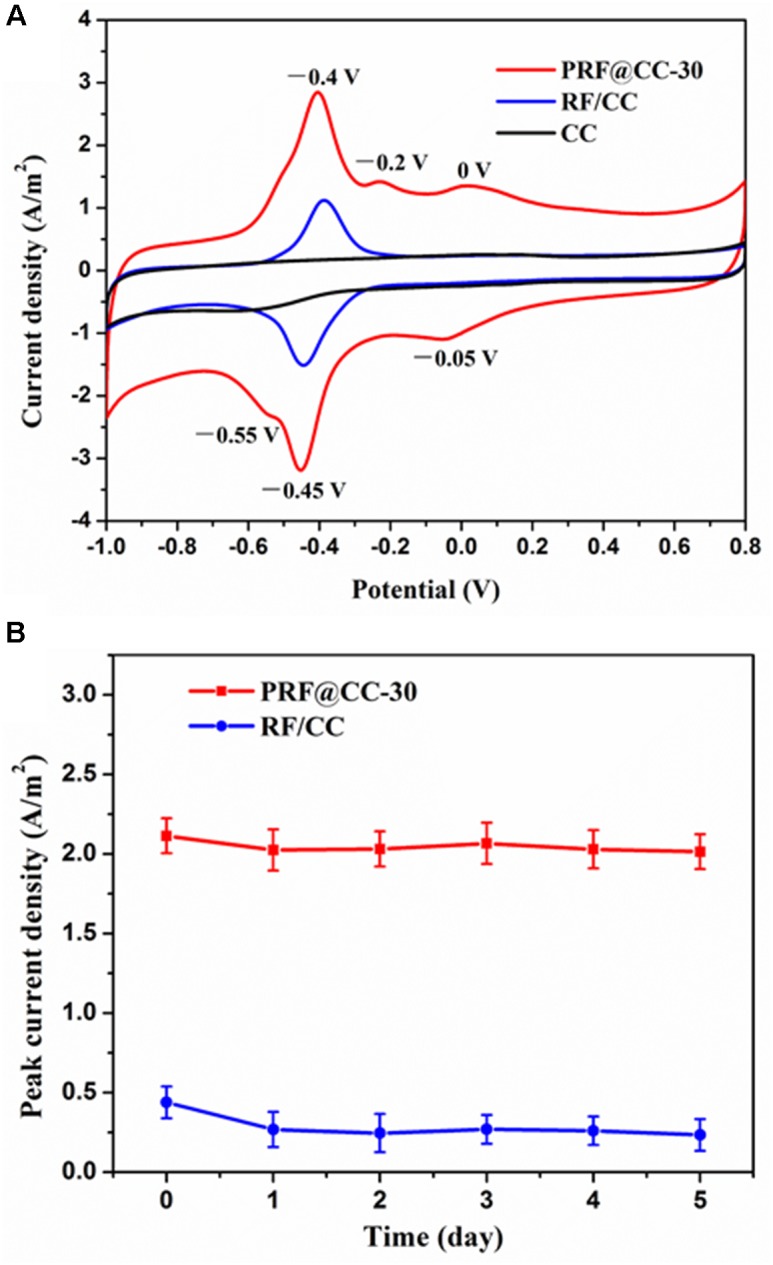
Electrochemical characterization of electrodes. **(A)** The CV curves of the PRF@CC-30, RF/CC and bare CC electrodes at a scanning speed of 30 mV/s in 0.1 M PBS solution, **(B)** and the variation of peak current density of riboflavin redox reaction for the PRF@CC-30 and RF/CC electrodes (each value is the mean of three replicates).

The existence of polyriboflavin on the CC electrode was further confirmed by electrochemical analysis (Figure 1A). The CV curves of both the PRF@CC-30 electrode and the RF/CC electrode in 0.1 M PBS solution revealed a clear redox pair of riboflavin with oxidation potential of -0.4 V and reduction potential of -0.45 V due to the redox process between the quinone and hydroquinone forms (Supplementary Figure S5), which is generally a two-electron transfer process (Chatterjee and Foord, 2009). In addition, the PRF@CC-30 electrode presented a pair of discernable redox waves with an oxidation potential of -0.2 V and a reduction potential of -0.55 V, which could be also originate from the two-electron electrochemical reactions due to the existence of deprotonated quinone and hydroquinone forms in small quantities in the neutral solution according to previous studies (Chatterjee and Foord, 2009; Radzevic et al., 2016). In fact, the above two-type redox reactions could also happen on the RF/CC electrode. However, there was another clear redox pair with an oxidation potential of 0 V and a reduction potential of -0.05 V in the CV curve of the PRF@CC-30 electrode, which has not been reported for the riboflavin monomers as far as we know. These newly appearing redox pair waves have been suggested to be attributed to the covalent binding between riboflavin molecules (Radzevic et al., 2016). Thus, the CV characteristic also verified the successful polymerization of riboflavin on the CC electrode, which endowed more flexible electrochemical activities and a faster interface electron transfer rate owing to the high loading amount of redox mediators. Subsequently, the stability of modifiers (either riboflavin monomers or polyriboflavin) grafted on the electrode, which is of great significance for practical applications, was evaluated by calculating peak current density of riboflavin redox reaction when the electrode was consistently immersed in 0.1 M PBS solution. As shown in Figure 1B, the peak current density for PRF@CC-30 electrode dropped by 4.21% within 1 day and then stabilized at a relatively high value, while almost 38.95% was lost for the RF/CC electrode. This result strongly illustrated a much better stability of the electrochemically polymerized riboflavin on the CC electrode surface than the physically adsorbed one.

### Polyriboflavin Interface Enhanced Outward EET for MFC Bioelectricity Production

**FIGURE 2:**
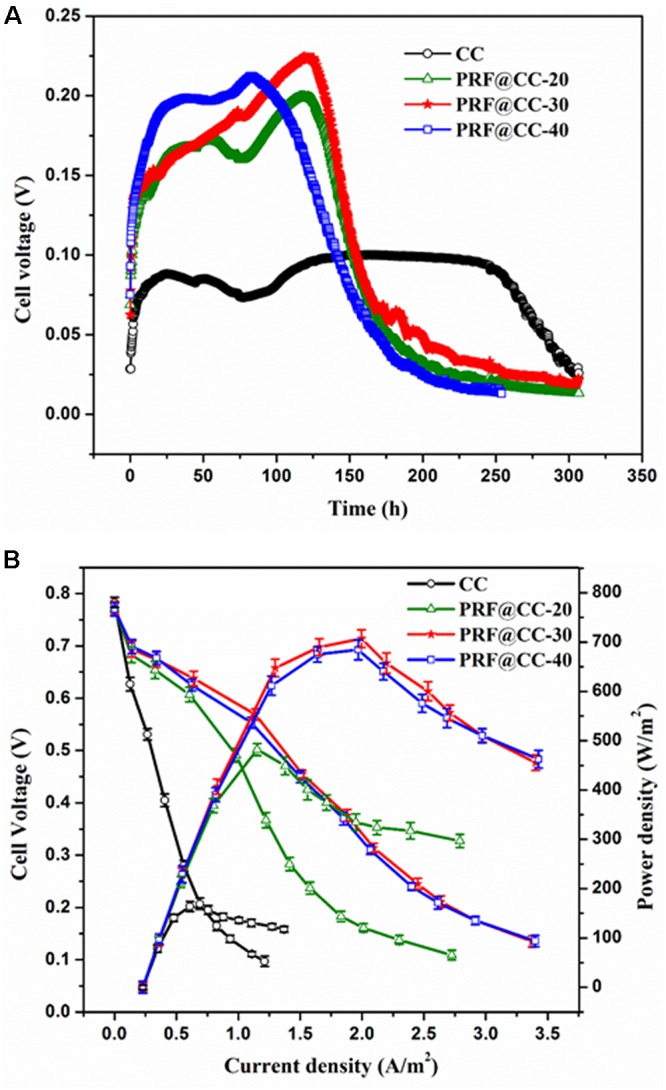
Bioelectricity production in dual-chamber MFCs equipped with different electrodes. **(A)** The output voltage curves under the external loading resistance of 1500 Ω, **(B)** the polarization curves and power density plots (each value is the mean of three replicates).

The effect of the polyriboflavin interface on *Shewanella* bioelectrocatalysis was first investigated in MFCs in terms of bioelectricity production. For comparison, the prepared PRF@CC electrodes with different degrees of electropolymerization and bare carbon cloth were applied as anodes for dual-chamber MFCs. As shown in Figure 2A, the output voltages of all MFCs equipped with PRF@CC electrodes increased rapidly and reached a high range from 0.175 to 0.225 V, while the voltage of CC-based MFC was not higher than 0.1 V. Although the start-up period was difficult to be defined in real MFCs due to many anfractuous influence factors, an increase in the start-up rate could be clearly observed for PRF@CC electrodes compared to bare CC electrodes. The PRF@CC-30 electrode, with an approved electricity production and a relatively moderate electropolymerization degree, was further evaluated for stability in the bioelectrocatalytic process, which showed four-cycle repeatable discharge platforms without obvious loss in output voltage (Supplementary Figure S6). The result indicated a reliable stability and durability for the prepared PRF@CC electrode, likely due to the strong interaction between polyriboflavin and carbon fibers of the CC substrate. Nevertheless, the enhancement of the polyriboflavin interface on the anodic bioelectrocatalysis in an MFC might still be underestimated because of the possible mismatch between the external load-resistance and the internal resistance of MFC (Zou et al., 2016a). Therefore, the polarization and power density curves (Figure 2B) were drawn by using a series of external resistances, which could visually indicate the maximum attainable power density achieved at proper match between the external load resistance and the internal resistance. Strikingly, the PRF@CC-30 anode delivered a maximum power density of 707 mW/m^2^, almost 4.3-fold higher than that of the CC anode (164 mW/m^2^). Although the achieved power density and voltage output for the PRF@CC-30 anode were relatively moderate, the magnification factor of power density for the CC electrode after electrochemical functionalization with polyriboflavin was comparable to those reported in other research when using bacterial strains of *Shewanella* species and under similar operating conditions (Supplementary Table S1). In consideration of their similar open-circuit voltages and the parallel cathodes, it is suggested that the higher bioelectricity generation of PRF@CC-30-based MFC is attributed to the improvement in anodic EET process and accompanies a great decrease in the internal resistance of MFC devices. This postulation was further validated by analyzing the *V*–*j* curve, in which the slope of linear potential drop is proportional to the internal ohmic resistance (Yu et al., 2015). Clearly, the lower slope in the *V*–*j* curve of MFC with PRF@CC-30 electrodes suggested a decreased internal ohmic resistance compared to the CC-based MFC. The internal resistance of an MFC could also be calculated roughly as the external load-resistance when the output voltage was half of the open-circuit voltage, so that the PRF@CC-30 based MFC had an internal resistance of about 1.61 kΩ, which was much lower than that of CC-based MFC (about 13.96 kΩ). Thus, the great improvement of PRF@CC electrodes in *Shewanella* bioelectrocatalysis for MFC bioelectricity generation was strongly attributed to the lowered internal resistance, in particular the ohmic resistance against anodic interfacial electron transfer from *Shewanella* cells to polyriboflavin interface, because of the same device configuration, inoculum, cathode and operational conditions for all MFCs.

**FIGURE 3:**
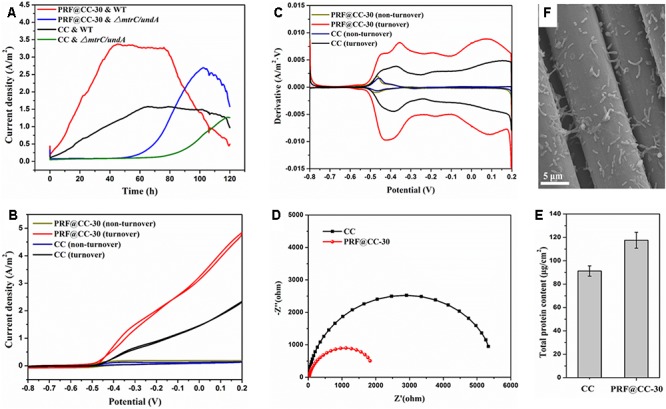
Electrochemical behaviors of the PRF@CC-30 and bare CC electrodes in three-electrode cells inoculated with either wild-type (WT) *Shewanella putrefaciens* CN32 or its *ΔmtrC/undA* mutant strain, and the microbial biofilm growth. **(A)** The amperometric I-T curves when a poised potential of +0.2 V, **(B)** the CV curves under either turnover condition (lactate consumption) or non-turnover condition (lactate depletion) and **(C)** the corresponding first-order derivatives, **(D)** the Nyquist plots under an initial potential of -0.45 V, **(E)** a representative SEM image of microbial cells grown on electrode and **(F)** the total protein content attached on electrodes.

To further elaborate the influence of the polyriboflavin interface on the anodic (outward) EET, the PRF@CC-30 electrode and CC electrode were analyzed for comparison in the three-electrode half-cell reactor that involves fewer impact factors and possesses better controllability. When poised at a potential of + 0.2 V, the PRF@CC-30 electrode delivered a high current density with a plateau around 3.31 A/m^2^ for about 40 h in a half-cell reactor inoculated with wild-type *S. putrefaciens* CN32, which dramatically preceded the CC electrode with a stable current density of approximately 1.57 A/m^2^ (Figure 3A). The start-up time to reach the plateau of PRF@CC-30 electrode (less than 40 h) is shorter than that of the CC electrode (almost 65 h), indicating the much faster start-up rate for the PRF@CC-30 electrode. The results are in agreement with the aforementioned findings in dual-chamber MFCs. Subsequently, in order to analyze the *Shewanella* EET pathways on the polyriboflavin interface, CVs (Figure 3B) of the PRF@CC-30 electrode and CC electrode were performed both during the plateau of current density and after the sharp downfall of current output, where the bioelectrocatalytic reaction for substrate consumption (i.e., turnover condition) and the substrate depletion (i.e., non-turnover condition) occurred, respectively. CVs of both the PRF@CC-30 electrode and CC electrode showed obvious catalytic current response under the turnover condition but not under the non-turnover condition, strongly demonstrating the bioelectrocatalytic activity toward lactate consumption. Notably, the CV of the PRF@CC-30 electrode exhibited a much higher bioelectrocatalytic current density with a more negative on-set potential and two-pair obvious redox waves in comparison with the CC electrode, which suggests a more favorable driving force and faster speed of EET from bacterial cells to the PRF@CC-30 electrode interface. According to the first-order derivatives of the above CVs (Figure 3C), the PRF@CC-30 electrode showed richer and larger redox waves than the CC electrode under the turnover condition in the potential range of higher than -0.6 V. However, no obvious difference under the non-turnover condition was observed, except for a slight change of peak potential and current density responsible for the riboflavin redox reaction. Considering the previously reported broad potential window of *Shewanella* membrane-bound cytochromes (Firer-Sherwood et al., 2008; Baron et al., 2009; Carmona-Martinez et al., 2011; Okamoto et al., 2011), it was suggested that the polyriboflavin interface not only can improve the MET but might well also promote the DET by means of a potential co-action of riboflavin and cytochromes. Strikingly, the PRF@CC-30 electrode delivered undetectable current response within the first 50 h when the *ΔmtrC/undA* mutant strain was used as an inoculum (Figure 3A) due to the crucial role of outer-membrane cytochromes in both DET and MET of *Shewanella* strains (Coursolle et al., 2010; Okamoto et al., 2011; Wu et al., 2018). The latter partial recovery might be owing to the increased cell membrane permeability for transporting mediators and/or the activation of other cytochromes for reducing mediators along with the progress of electrochemical acclimation (Wu et al., 2018). The Nyquist plots (Figure 3D) were conducted to analyze the interfacial charge-transfer resistance (*R_ct_*), which clearly demonstrated a much smaller *R_ct_* of the PRF@CC-30 electrode. Thus, it substantially proved the above-mentioned hypothesis that the polyriboflavin functionalization decreased the ohmic resistance against anodic interfacial electron transfer. After one cycle of amperometric I-T testing, the microbes grown on an electrode were successively immobilized using 4 wt% glutaraldehyde solution and dehydrated with increasing concentrations of ethanol solution for SEM observation (Figure 3E). Although no distinct difference in bacterial loading was observed by SEM, the total protein content attached to the PRF@CC-30 electrode was statistically higher than that to the CC electrode (Figure 3F), indicating the great biocompatibility of polyriboflavin interface.

### Polyriboflavin Interface Promoted Inward EET for Fumarate Reduction

Besides outward EET for anodic bioelectricity generation, *Shewanella* species have the ability to utilize an electrode (cathode) as electron donors for fumarate reduction by virtue of inward EET approach. The Mtr respiratory pathway in *S. oneidensis* MR-1 has been demonstrated to be functionally reversible and was able to conduct the inward electron flux from an electrode into periplasmic reductase for intracellular fumarate reduction (Ross et al., 2011). In addition, an increase in riboflavin concentration in electrolytes also dramatically improved the cathodic current consumption for fumarate reduction in *S. oneidensis* MR-1 (Yang et al., 2015; Si et al., 2016). Thus, these findings compel further study on the effect of polyriboflavin interface on inward EET processes and bioelectrocatalytic reduction of fumarate in *Shewanella*.

**FIGURE 4:**
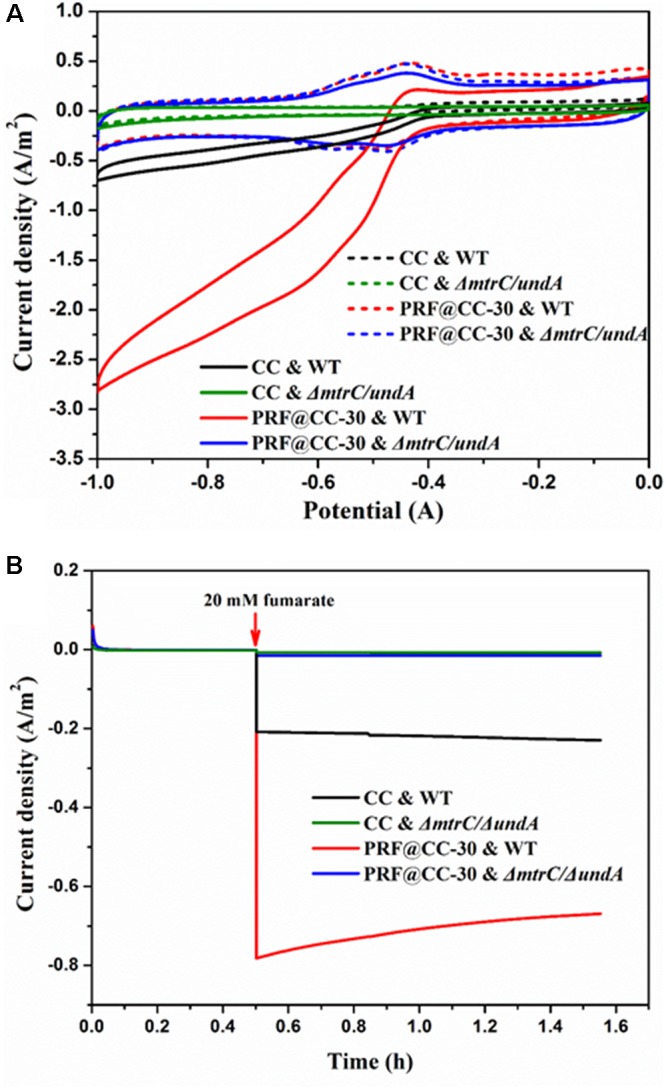
Bioelectrocatalytic fumarate reduction for the PRF@CC-30 and bare CC electrodes in three-electrode cells. **(A)** The CV curves of fumarate responses for different electrodes and inoculums (dashed line, no addition; solid line, 20 mM fumarate), **(B)** the amperometric I-T curves where electrodes were poised at –0.6 V and 20 mM fumarate was added after 30 min.

To confirm the ability of *S. putrefaciens* CN32 for fumarate reduction, CV analysis was first performed in the presence and absence of fumarate (Figure 4A). In an anaerobic three-electrode electrochemical cell inoculated with wild-type strain, CV curves of both the PRF@CC-30 and CC electrodes presented a clear reduction of current in the presence of 20 mM fumarate but not in the absence of fumarate, which is in accordance with the findings when using *S. oneidensis* MR-1 as a biocatalyst (Ross et al., 2011). Strikingly, the larger reduction current response for the PRF@CC-30 electrode indicated its faster fumarate reduction rate. However, both CV curves of the two electrodes delivered undetectable reduction current in an electrochemical cell inoculated with *ΔmtrC/undA* mutant strain. According to previous reports (Si et al., 2015, 2016) and the CV results, a reducing potential of -0.6 V was employed to enable fumarate reduction in the following amperometric tests. In anaerobic three-electrode cells inoculated with wide-type *S. putrefaciens* CN32 strain, continuous amperometry measurements (Figure 4B) for both the PRF@CC-30 electrode and the CC electrode showed a sudden onset of cathodic current upon addition of 20 mM fumarate. Impressively, the cathodic current density for the PRF@CC-30 electrode (∼0.78 A/m^2^) was almost 3.7-fold higher than that for the CC electrode (∼0.21 A/m^2^), which indicated a much higher inward EET rate for the PRF@CC-30 electrode, since the observed negative current was indicative of electron flow from the electrode into microbial cells. The slight decrease in cathodic current density for the PRF@CC-30 electrode as time prolongs could be ascribed to the gradual reduced fumarate concentration near the electrode. This is because of the fast consumption of substrate and the limited diffusion rate under non-stirring conditions. Furthermore, the *ΔmtrC/undA* mutant strain was used as an inoculum to analyze the role of outer-membrane cytochromes in the inward EET rate. Clearly, both electrodes showed negligible cathodic current signals in response to the addition of fumarate. This result proved the essential role of outer-membrane cytochromes in the inward EET pathway and the substantial function of the polyriboflavin interface on accelerating electron transfer from an electrode to outer-membrane cytochromes of the *Shewanella* strain.

## Conclusion

In summary, this work for the first time demonstrated the great promotion effect of an electropolymerized polyriboflavin interface on the *Shewanella* bidirectional EET rate for high bioelectrocatalytic ability. Particularly, the optimal PRF@CC electrode achieved almost 4.3-fold increased output power density in dual-chamber MFCs and 3.7-fold cathodic current density for fumarate reduction in three-electrode cells in comparison with the bare CC electrode, respectively. In addition, the great increases in anodic electricity production and cathodic fumarate reduction were also observed for the polyriboflavin-functionalized graphene electrode (Supplementary Material, Section II, Supplementary Figures S7–S10). The significantly increased EET process may well be attributed to the fast electron exchange between the polyriboflavin interface with flexible electrochemical activity and good biocompatibility and the outer-membrane cytochromes of the *Shewanella* strain. Taking into consideration the fact that riboflavin is widely involved in the EET pathways of various microorganisms (both Gram-positive and Gram-negative ones) (Okamoto et al., 2014; Jia et al., 2017; Light et al., 2018; You et al., 2018), together with the simple fabrication approach and reliable electrocatalytic ability, the functional polyriboflavin interface is expected to hold promising potential in more applications such as environmental remediation and biosensing.

## Author Contributions

LZ and Z-eL designed the experiments. LZ and XW performed the experiments. YH and HN contributed to analyze the experiment data. LZ wrote the manuscript. Z-eL revised the manuscript.

## Conflict of Interest Statement

The authors declare that the research was conducted in the absence of any commercial or financial relationships that could be construed as a potential conflict of interest.
