# Supplementary material for: Promoting Shewanella Bidirectional Extracellular Electron Transfer for Bioelectrocatalysis by Electropolymerized Riboflavin Interface on Carbon Electrode
Source: Front Microbiol. 2019 Jan 15;9:3293. doi: 10.3389/fmicb.2018.03293 (PMC6340934; doi:10.3389/fmicb.2018.03293)
Supplement: Supplementary file 1 [file Data_Sheet_1.pdf]

Supporting Information

Promoting *Shewanella* Bidirectional Extracellular Electron Transfer for Bioelectrocatalysis by Electropolymerized Riboflavin Interface on Carbon Electrode

Long Zou*[†]*, Xian Wu*[†]*, Yunhong Huang, Haiyan Ni and Zhong-er Long*

College of Life Science, Jiangxi Normal University, Nanchang, China

[*]Correspondence: Zhong-er Long, longzhonger@163.com

[†]These authors have contributed equally to this work

Section I

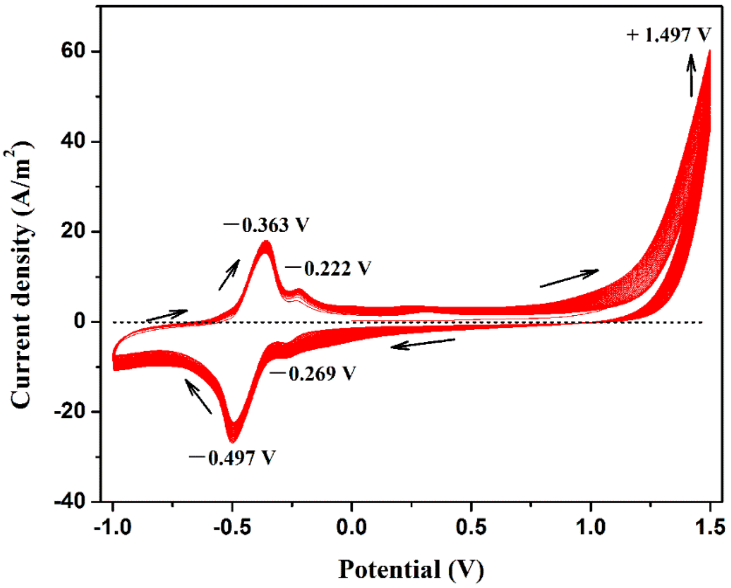

Figure S1 Electropolymerization CV at a scanning speed of 50 mV/s for 1 mM riboflavin in 0.1 M PBS solution with the addition of 0.15 M NaCl (pH 7.0). Arrows show the direction of peak the changes during the potential cycling.

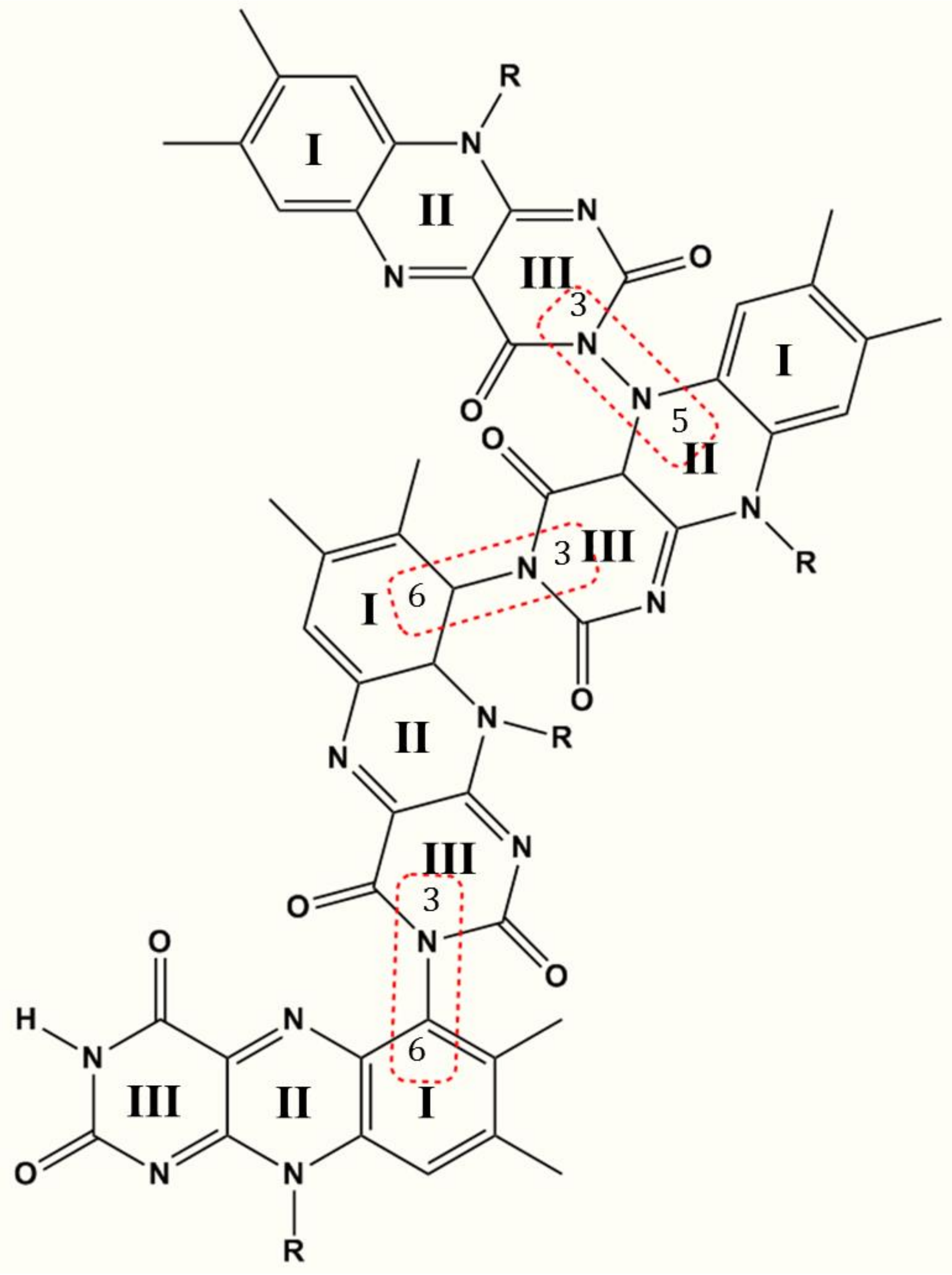

Figure S2 Presentation of possible bonding mode of polyriboflavin. The possible bounds are highlighted by red dotted box. R is for D-ribityl. It was redrew according to a previous report (Radzevic et al., 2016).

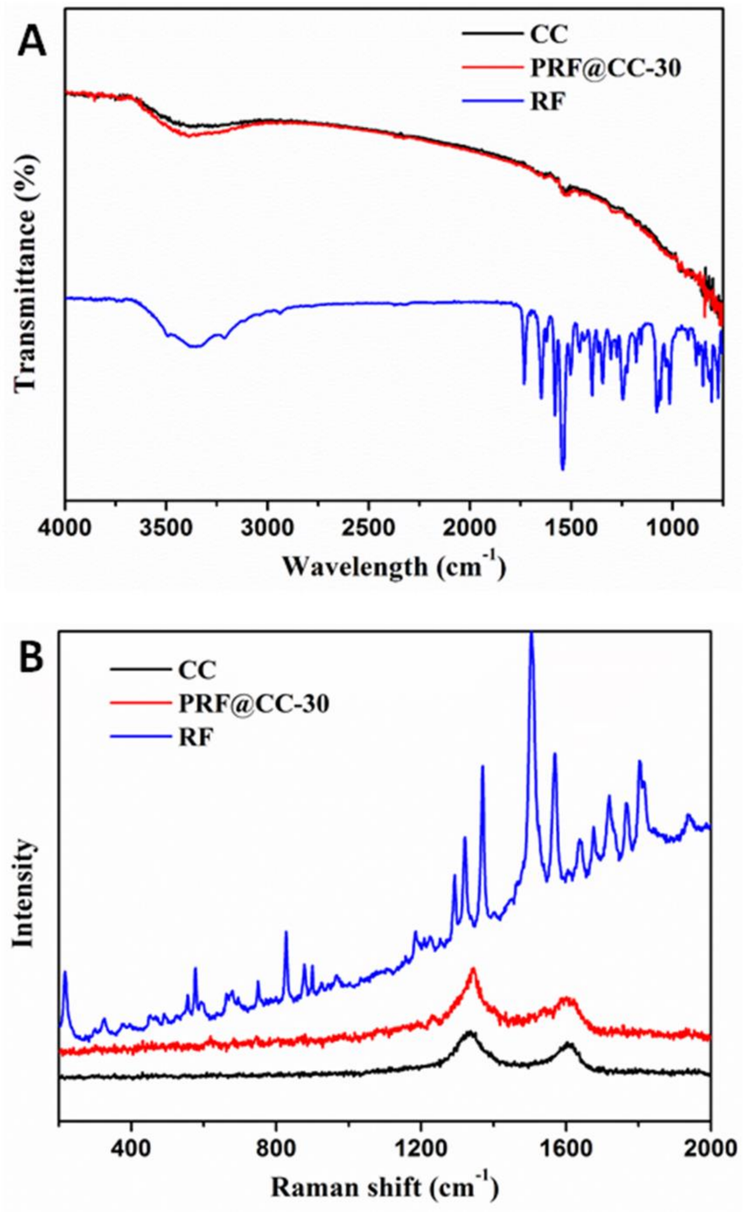

Figure S3 The fourier transform infrared (FTIR) spectra (A) and Raman spectra (B) of the PRF@CC-30 electrode, CC electrode and pure riboflavin.

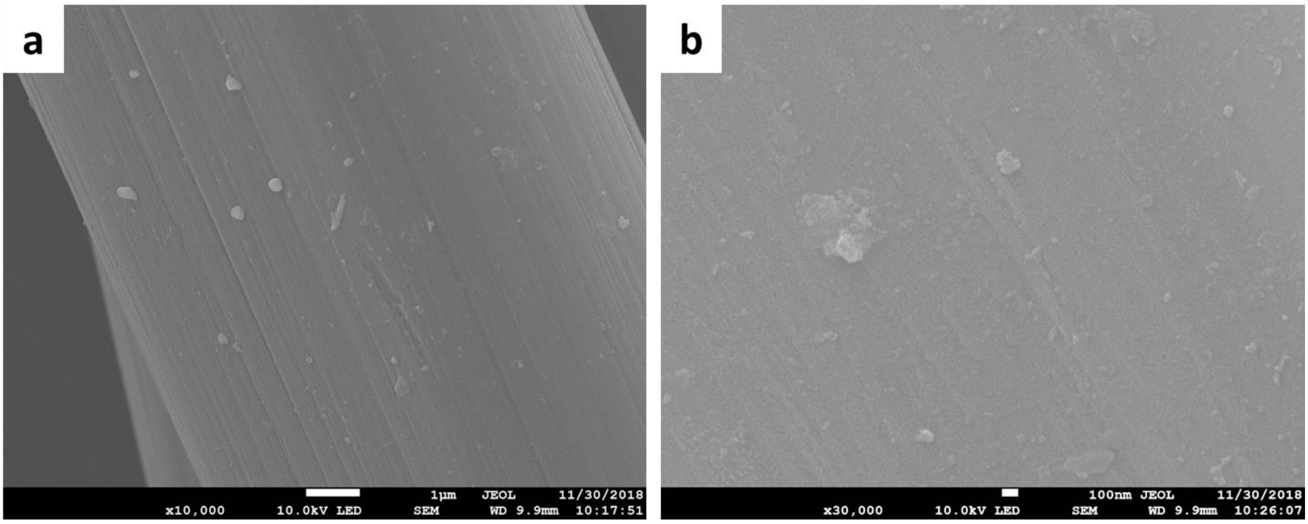

Figure S4 The SEM images in different resolution of polymerized riboflavin thin film on carbon fiber.

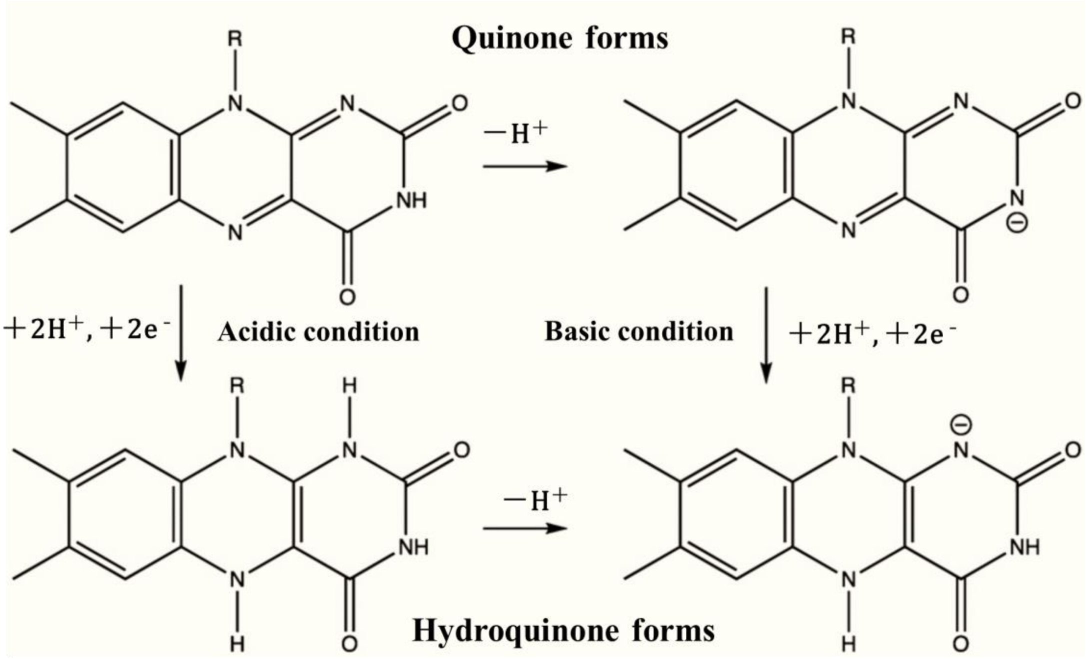

Figure S5 Schematic diagram of redox reaction between quinone form and hydroquinone form of riboflavin. R is for D-ribityl. It was redrew according to a previous report (Chatterjee and Foord, 2009).

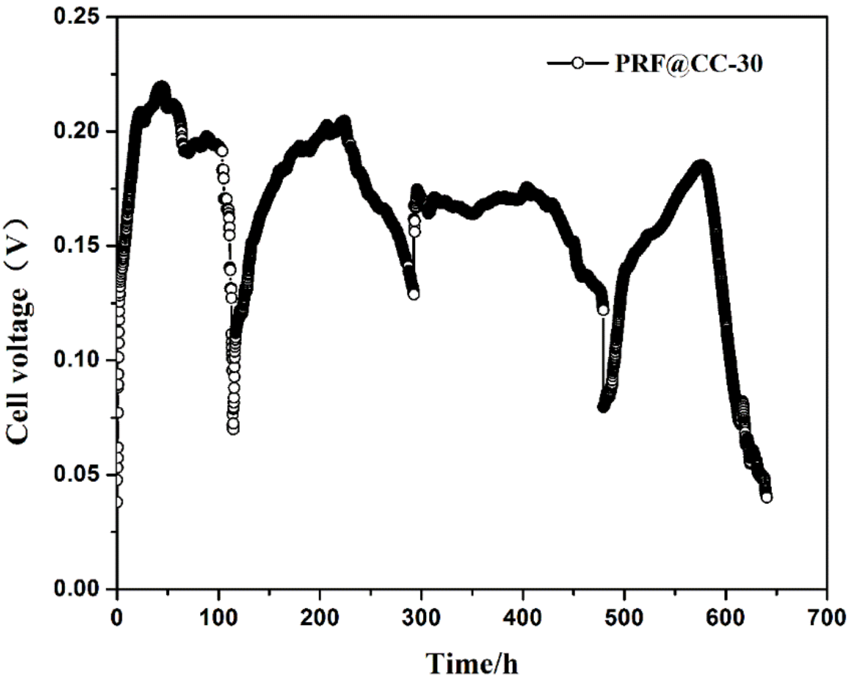

Figure S6 The four-cycle output voltage of the MFC equipped with PRF@CC-30 anode.

Table S1 Summary of the recently reported performance of MFCs inoculated with *Shewanella* species under similar operating conditions.

**Electrode**	**Inoculum**	**Power density (mW/m^2)**	**Magnification factor**	**Reference**
CC (carbon cloth)	*S. putrefaciens* CN32	164		This work
PRF@CC-30		707	4.31	
CF (carbon felt)	*S. putrefaciens* CN32	215		(Zou et al., 2017)
Mo_2C@CF		1025	4.77	
CC	*S. putrefaciens* CN32	127		(Wu et al., 2018)
PC/Si/CC		580.7	4.57	
CC	*S. loihica* PV-4	98		(Liu et al., 2015)
PEDOT/CC		140	1.43	
CC	*S. oneidensis* MR-1	51.5		(Liu et al., 2017)
PANI/CC		388.6	7.5	
GP (graphite paper)	*S. oneidensis* MR-1	41		(Wang et al., 2017)
GP/RF		148	3.6	
GO/RF		257	6.3	
CF	*S. oneidensis* MR-1	190		(Hasan et al., 2017)
NQ-LPEI/CF		530	2.79	

Section II: ***Polyriboflavin interface on graphene modified CC electrode***

The role of polyriboflavin interface in microbial electrocatalysis was further investigated on a graphene modified CC (G/CC) electrode prepared by a facile two-step electrochemical deposition. The electrochemical deposition of graphene onto CC electrode was carried out in a two-electrode electrochemical cell with a platinum sheet as the counter electrode and 0.2 mg/mL graphene oxide (GO) solution as the electrolyte. According to a previous report (Liu et al., 2012), the negative-charged GO was electrophoretically transported onto CC electrode surface by using 0.3 mA/cm^2 anodic current for 15 min, followed by 0.6 mA/cm^2 cathodic current for 90 s to reduce GO to graphene (**Figure S7**). The modification of G/CC electrode with polyriboflavin was also obtained through the same electropolymerization procedure as PRF@CC electrodes, but with different CV cycles from 30 to 60. The prepared electrodes were designated as PRF@G/CC-30, PRF@G/CC-40, PRF@G/CC-50 and PRF@G/CC-60, respectively.

As shown in **Figure S8**, the G/CC-base MFC delivered a much larger output voltage (over 0.2 V) than that of CC-based MFC, which is in good agreement with a previous study (Liu et al., 2012). More impressively, the MFC output voltage still achieved an increase to a great extent with the help of the interfacial functionalization of G/CC electrode with polyriboflavin, accompanying with a faster start-up rate. Furthermore, all the polyriboflavin-functionalized G/CC electrodes also showed much higher current densities and faster start-up rate than the G/CC electrode in three-electrode electrochemical cells where the working electrodes under a fixed potential of + 0.2 V. Compared to the G/CC electrode, the PRF@G/CC-50 electrode a more favorable driving force and faster EET speed in three-electrode half-cell reactor (**Figure S9**). In addition, the polyriboflavin functionalized G/CC electrode also achieved a great enhancement on cathodic fumarate reduction in comparison with the G/CC electrode (**Figure S10**). These results substantially demonstrated the promising potential of electropolymerized riboflavinint erface in interfacial functionalization of different carbon electrodes for boosting both anodic and cathodic bioelectrocatalysis performance.

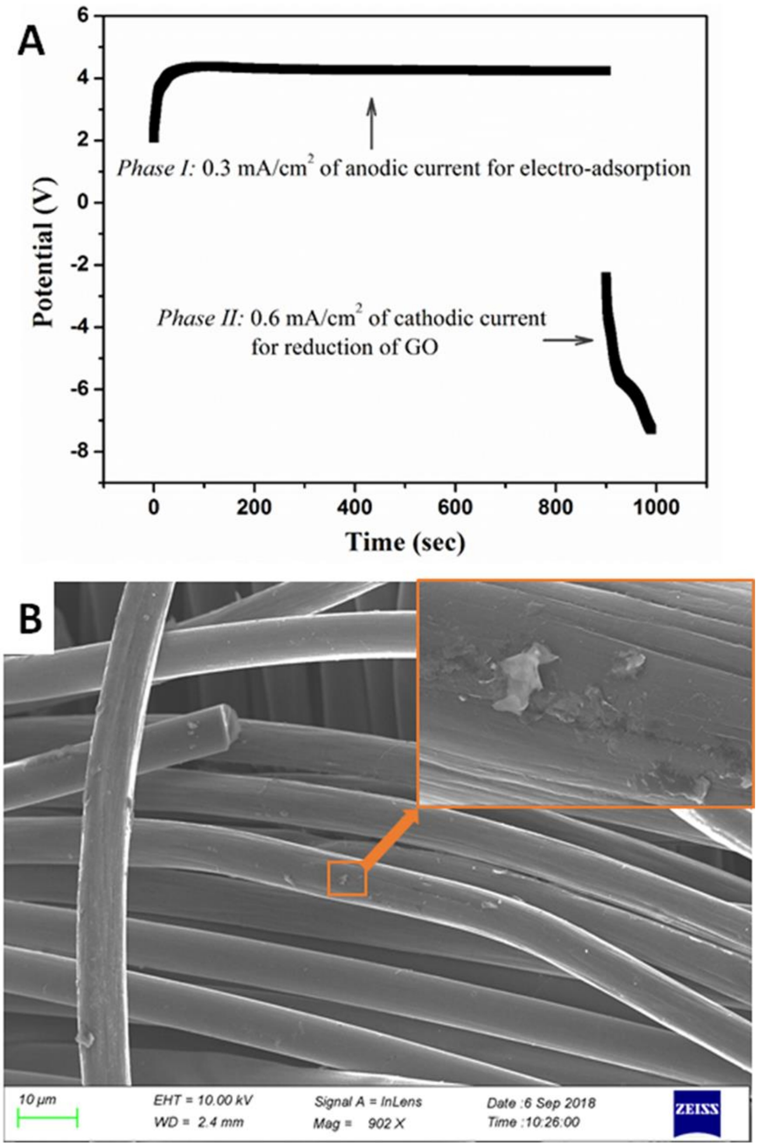

Figure S7 The constant-current electrochemical deposition of graphene onto CC electrode in a two-electrode cell (A) and the SEM image of prepared G/CC electrode (B).

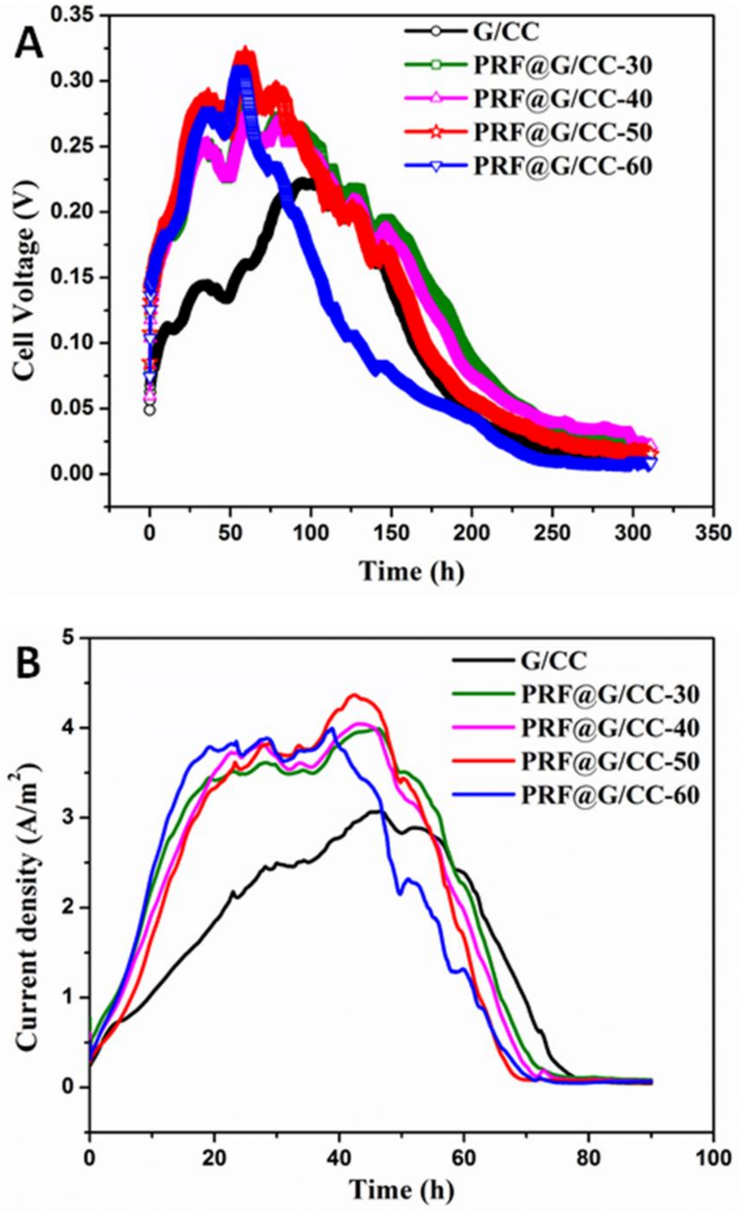

Figure S8 The output voltage curves of MFCs equiped with different anodes under a external load-resistance of 1500 Ω (A) and the amperometric I-T curves of different electrode posited at a potential of + 0.2 V in three-electrode cells (B).

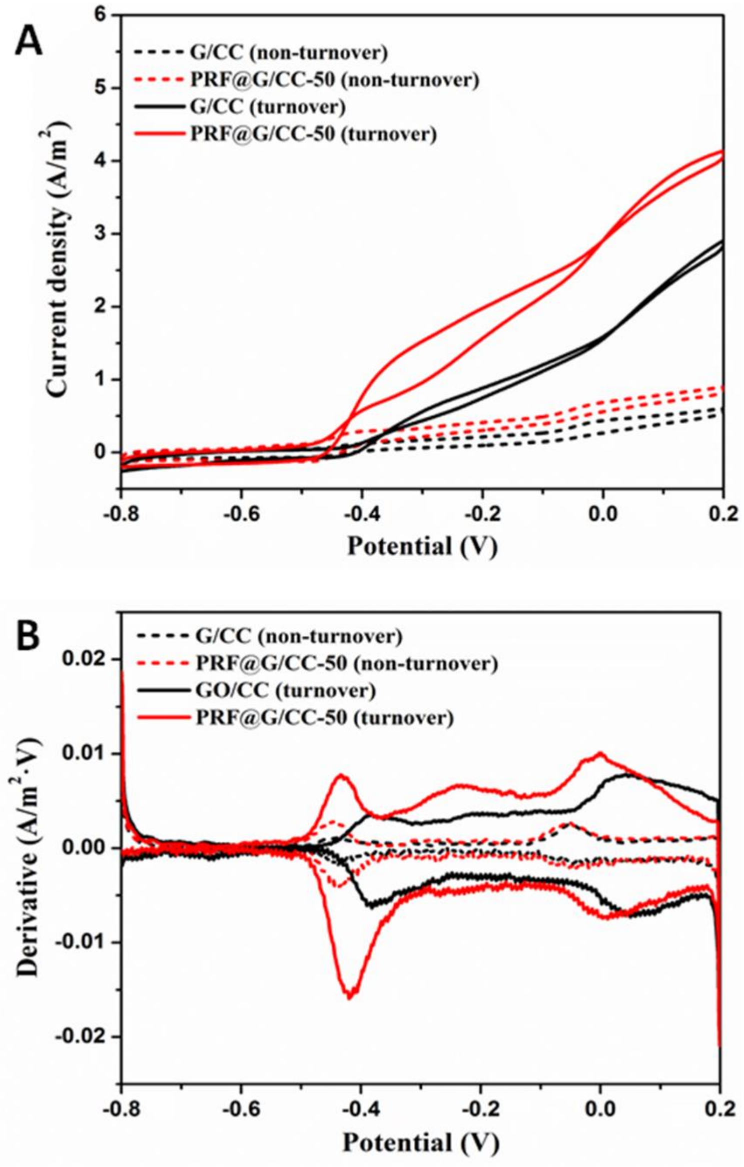

Figure S9 The CV curves of PRF@G/CC-50 electrode and G/CC electrode under either turnover condition (substrate consumption) or non-turnover condition (substrate deleption) (A), and the corresponding first-order derivatives (B) in three-electrode cells. ***Note:*** *CVs of both two electrodes showed remarkable current response with two-pair obvious redox waves under the turnover condition but negligible response under the non-turnover condition, indicating the bioelectrocatalytic activity towards lacate consumption. Obviously, the CV of PRF@G/CC-50 electrode exhibited much higher bioelectrocatalytic current density with more negative on-set potential compared to the G/CC electrode, which suggests a more favorable driving force and faster EET speed. Additionally, compared to the G/CC electrode, the PRF@G/CC-50 electrode showed similar but larger redox waves under the turnover condition in the potential range of higher than – 0.55 V, as shown in the corresponding first-order derivative curves. The above results are in good agreement with that of polyriboflavin functionalization of CC electrode.*

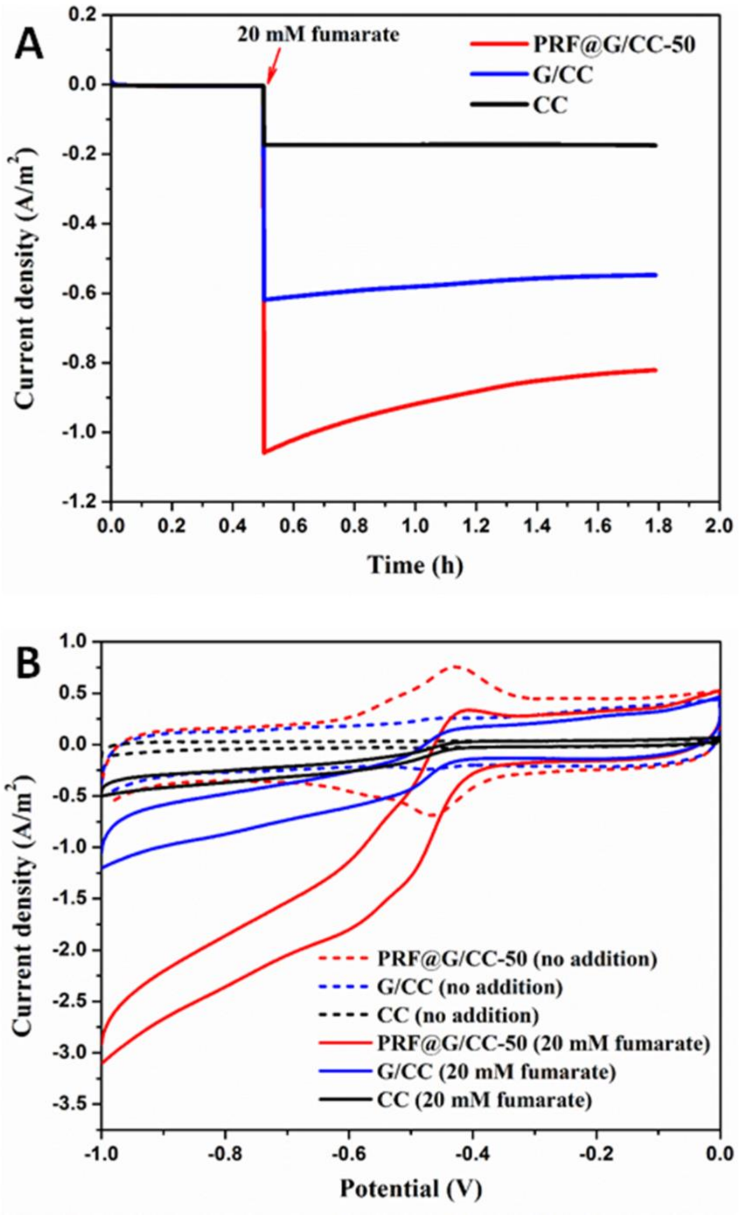

Figure S10 The bioelectrocatalytic fumarate reduction for the PRF@G/CC-50 and G/CC electrodes in three-electrode cells. (A) Amperometric I-T curves where electrodes were poised at – 0.6V and 20 mM fumarate was added after 30 min, (B) CV curves of fumarate responses for different electrodes and inoculums (dashed line, no addition; solid line, 20 mM fumarate).

Refrences

Chatterjee, A., and Foord, J.S. (2009). Biological applications of diamond electrodes; electrochemical studies of riboflavin. *Diam. Relat. Mater.* 18(5)**,** 899-903. doi: 10.1016/j.diamond.2009.02.016.

Hasan, K., Grattieri, M., Wang, T., Milton, R.D., and Minteer, S.D. (2017). Enhanced

bioelectrocatalysis of *Shewanella oneidensis* MR-1 by a naphthoquinone redox polymer. *ACS Energy Lett.* 2(9)**,** 1947-1951. doi: 10.1021/acsenergylett.7b00585.

Liu, J., Qiao, Y., Guo, C.X., Lim, S., Song, H., and Li, C.M. (2012). Graphene/carbon cloth anode for high-performance mediatorless microbial fuel cells. *Bioresource Technol.* 114**,** 275-280. doi: 10.1016/j.biortech.2012.02.116.

Liu, X., Wu, W., and Gu, Z. (2015). Poly (3,4-ethylenedioxythiophene) promotes direct electron transfer at the interface between *Shewanella loihica* and the anode in a microbial fuel cell. *J. Power Sources* 277**,** 110-115. doi: 10.1016/j.jpowsour.2014.11.129.

Liu, X., Zhao, X.H., Yu, Y.Y., Wang, Y.Z., Shi, Y.T., Cheng, Q.W., et al. (2017). Facile fabrication of conductive polyaniline nanoflower modified electrode and its application for microbial energy harvesting. *Electrochim. Acta* 255**,** 41-47. doi: 10.1016/j.electacta.2017.09.153.

Radzevic, A., Niaura, G., Ignatjev, I., Rakickas, T., Celiesiute, R., and Pauliukaite, R. (2016). Electropolymerisation of the natural monomer riboflavin and its characterisation. *Electrochim. Acta* 222**,** 1818-1830. doi: 10.1016/j.electacta.2016.11.166.

Wang, Q.-Q., Wu, X.-Y., Yu, Y.-Y., Sun, D.-Z., Jia, H.-H., and Yong, Y.-C. (2017). Facile *in-situ* fabrication of graphene/riboflavin electrode for microbial fuel cells. *Electrochim. Acta* 232**,** 439-444. doi: 10.1016/j.electacta.2017.03.008.

Wu, X., Qiao, Y., Shi, Z., and Li, C.M. (2018). Enhancement of interfacial bioelectrocatalysis in *Shewanella* microbial fuel cells by a hierarchical porous carbon-silica composite derived from distiller's grains. *Sustain. Energy Fuels* 2(3)**,** 655-662. doi: 10.1039/C7SE00560A.

Zou, L., Lu, Z., Huang, Y., Long, Z.-e., and Qiao, Y. (2017). Nanoporous Mo_2C functionalized 3D carbon architecture anode for boosting flavins mediated interfacial bioelectrocatalysis in microbial fuel cells. *J. Power Sources* 359**,** 549-555. doi: 10.1016/j.jpowsour.2017.05.101.
